# Sex-Specific transcriptomic changes in adipose tissue following adult-onset disruption of growth hormone receptor

**DOI:** 10.1007/s11102-025-01603-3

**Published:** 2025-12-05

**Authors:** Silvana Duran-Ortiz, Jonathan A. Young, Edward O. List, Reetobrata Basu, Christopher Walsh, Emmanuel A. Gotte, Darlene E. Berryman, John J. Kopchick

**Affiliations:** 1 Institute for Molecular Medicine and Aging, Heritage College of Osteopathic Medicine, Ohio University, Athens, OH USA; 2 Molecular and Cellular Biology Program, Ohio University, Athens, OH USA; 3 Department of Biological Sciences, College of Arts and Sciences, Ohio University, Athens, OH USA; 4 Department of Biomedical Sciences, Heritage College of Osteopathic Medicine, Ohio University, Athens, OH USA

**Keywords:** Growth hormone, Aging, Adipose tissue, RNAseq, 6mGHRKO mice, Extracellular matrix (ECM) remodeling

## Abstract

Reduction in growth hormone (GH) signaling throughout life is known to extend lifespan and enhance healthspan in mice, and congenital GH receptor (GHR) mutations in both mice and humans confer protection against age-related diseases such as cancer, diabetes, and cognitive decline. To explore the health effects of disrupting GH action during adulthood, we previously generated adult-onset GHR knockout (6mGHRKO) mice by ablating GHR at 6 months of age. Both male and female 6mGHRKO mice exhibited reduced oxidative stress, with males showing improved insulin sensitivity and resistance to cancer, while females demonstrated extended lifespan. In the current study, we performed RNA sequencing on subcutaneous adipose tissue (Subq AT) from 6mGHRKO and control mice to investigate molecular mechanisms underlying these health benefits. Differential gene expression, gene ontology, pathway enrichment, and upstream regulator analyses revealed that GHR ablation predominantly downregulated gene expression, particularly in males. Sex-specific gene expression differences were more pronounced in control mice than in 6mGHRKO counterparts. Among the enriched processes, pathways related to extracellular matrix (ECM) organization emerged as differentially regulated between sexes and genotypes. These transcriptomic findings are exploratory and hypothesis-generating, highlighting ECM remodeling as a potential area for future mechanistic validation.

**Supplementary Information:**

The online version contains supplementary material available at 10.1007/s11102-025-01603-3.

## Introduction

GH is primarily secreted by the anterior pituitary gland and has a wide range of physiological effects including promoting somatic growth. GH action on target tissues also stimulates the production of another potent growth factor, Insulin-like growth factor-1 (IGF-1). Therefore, many physiological effects of GH are also mediated by IGF-1 (aka GH/IGF-1 axis) [1]. One of the primary targets of GH is adipose tissue (AT). Besides stimulating IGF-1 production in AT, GH promotes lipolysis, increasing circulating free fatty acids, which may contribute to its diabetogenic effects [2]. As a metabolically active organ, AT plays a key role in systemic aging through adipokines, inflammation, and energy balance—processes influenced by the GH/IGF-1 axis. Accordingly, studies across various organisms have shown that reducing the GH/IGF-1 axis can enhance long-term health and increase lifespan [3]. In humans, extensive studies in Ecuadorians with Laron Syndrome (LS), caused by germline inactivating mutations in the GH receptor (GHR), demonstrate improved insulin sensitivity, resistance to diabetes and cancer, enhanced memory function, and decreased pro-aging signaling [4].

The mouse model of LS was developed in our laboratory and is known as the GH receptor knockout (GHRKO) mouse [5]. These animals have a germline GHR deletion, which results in a healthy longevity phenotype [6]. That is, GHRKO mice have reduced body size, improved insulin sensitivity, resistance to diet-induced diabetes, lower cancer incidence, and better cognitive function in later life. Importantly, these mice are relatively obese—despite their extended longevity—with a preferential expansion of subcutaneous fat [7], elevated adiponectin levels [8], decreased AT senescence [9], and altered immune cell profiles within adipose tissue [10]. The combination of these phenotypic traits establishes GHRKO mice as a model of healthy aging, earning them the Methuselah Mouse Prize as the longest-lived laboratory mice to date. [10.1038/news030915-13]. Furthermore, these mice display fewer aging markers, such as, reduced mTORC1 signaling in the liver, kidney, heart, and muscle [9, 11], and higher expression of genes associated with resistance to oxidative stress in the liver [12, 13]. These findings suggest that inhibiting the GH/IGF-1 axis could be a promising pharmacological strategy to promote healthy aging [14].

To investigate if inactivation of GHR at timepoints after birth can replicate the health benefits mentioned above for GHRKO mice, our laboratory has developed two mouse models with GHR disruption at different stages after birth: at pubertal age (1.5 months, referred to as 1.5mGHRKO mice) [15] and at mature adult age (6 months, referred to as 6mGHRKO mice) [16], which correspond to approximately 15 and 30 years in humans, respectively [ISBN: 978-0-12–369455-3]. These mice allow us to study the effects of decreased GH action at specific time points while preserving the beneficial action of GH to promote somatic growth into adulthood. Our research indicates that postnatal GHR disruption leads to sex-specific lifespan effects, with both 1.5mGHRKO and 6mGHRKO females living significantly longer than control mice [15, 16]. Recently, we also reported that 6mGHRKO mice exhibit reduced lean mass and increased fat mass, specifically in the Subq AT depot [16]. They also show improved health markers, including enhanced insulin sensitivity and reduced cancer incidence in males, as well as decreased kidney damage and lipid and protein oxidation in the liver and AT of both male and female 6mGHRKO mice [16].

AT, particularly Subq AT, undergoes significant changes in response to the GH/IGF-1 axis. Therefore, understanding these changes is crucial for comprehending the health benefits observed in mice with reduced GH action. To investigate potential mechanisms responsible for the health benefits seen in mice with postnatal ablation of the GHR, this study utilized RNA sequencing (RNAseq) to explore the molecular mechanisms and cellular pathways altered in the AT of male and female 6mGHRKO mice compared to their wild-type counterparts. Given that the lifespan benefits observed in 6mGHRKO mice are sex-dependent, our study conducted two broad analyses: a genotype comparison and a sex comparison. For the genotype comparison, we aimed to understand the effect of postnatal GHR ablation by comparing adipose gene expression in male and female 6mGHRKO mice to that in the corresponding control mice. For the sex comparison, we sought to understand how postnatal GHR ablation differentially affects male and female mice by comparing gene expression between sexes within the same genotype.

Here, we present the gene expression profiles, biological processes, molecular mechanisms, and cellular pathways that may underly the lifespan and health advantages observed in mice with reduced GH action at a mature adult age.

## Materials and methods

### 6mGHRKO generation

Mice with a C57BL/6J genetic background carrying loxP sites flanking exon 4 of the Ghr gene were previously produced by the Knockout Mouse Project (KOMP) [17]. C57BL/6J mice expressing a ubiquitous Cre recombinase gene driven by the ROSA26 gene promoter/enhancer (B6.129-Gt(ROSA)26Sortm1(cre/ERT2)Tyj/J mice) were purchased from The Jackson Laboratory [15, 18]. Homozygous mice for both the floxed *Ghr* gene and the Cre recombinase gene were used to generate the 6mGHRKO mice as previously described [18]. Briefly, mice of 6 months of age were used to generat the 6mGHRKO and control mice by injecting Tamoxifen or peanut oil, respectively, for 5 consecutive days [18]. Mice were housed in a temperature and humidity-controlled room at 22 °C under a 14-hour light, 10-hour dark cycle, with 3–4 mice per cage, and with ad libitum access to water and standard laboratory chow (ProLab RMH 3000). All mouse protocols were approved by Ohio University’s Institutional Animal Care and Use Committee.

### Body composition and length

A Bruker Minispec NMR analyzer (Bruker Corp., The Woodlands, TX) was used to measure body composition at the time of dissection, (12 months of age, suppl. Table 1), as previously described [18]. Longitudinal body composition and body weight determined every month starting 1 day before Tam treatment until the time of dissection was described in a previous publication [18]. Body-length was measured at the time of dissection from the tip of the nose to the anus.

### Adipose collection and RNA extraction

Twelve-month old mice were euthanized by cervical dislocation, and adipose samples were dissected and weighed. For RNA isolation, tissue was flash frozen in liquid nitrogen and stored at −80 °C until further processing. Frozen tissues were homogenized using a Precellys 24-Dual homogenizer, and RNA isolated using the QIAGEN RNeasy Lipid Tissue Mini Kit following manufacturer’s instructions. The quantity and quality of total RNA were measured with the NanoDrop 2000c (Thermo Scientific). The RNA used for subsequent experiments had a 260/280 and a 260/230 ratio ≥ 1.8 to ensure purity and quality of the RNA. The quantity and quality of the isolated RNA were determined by the Agilent 2100 Bioanalyzer from Agilent Technologies at the Genomics Facility (Ohio University). The RNA used for RNA-seq had an RNA Integrity Number ≥ 8.

### RNA sequencing and differential gene expression analysis

Male and female adipose mRNA samples isolated from three 6mGHRKO mice of each sex and three control littermates of each sex were used to create cDNA libraries. First, mRNA was isolated from the total RNA samples with Takara Clontech SMART technology, according to the manufacturer’s protocol. Subsequently. the samples were indexed and sequenced on an Illumina NovaSeq S4. Basecalls and demultiplexing were performed with Illumina’s bcl2fastq software and a custom python demultiplexing program with a maximum of one mismatch in the indexing read for pooled samples. Alignment of the RNA-seq reads was performed using Ensembl top-level assembly with STAR. Subread: featureCount was used to generate gene counts derived from the number of uniquely aligned unambiguous reads. Sequencing performance was assessed for the total number of aligned reads, total number of uniquely aligned reads, and features detected. RSeQC was used to quantify the ribosomal fraction, known junction saturation, and read distribution over known gene models. It is important to note that RNA from each individual mouse (*n* = 3 per sex/genotype) was sequenced independently. No physical pooling of RNA occurred at the experimental stage. The term “pooling” in this study refers exclusively to the statistical integration of data during downstream bioinformatics analysis (edgeR/limma with voom normalization), where replicate samples were modeled together to increase statistical power and reduce variability.

All gene counts were then imported into the R/Bioconductor package EdgeR and TMM normalization size factors were calculated to adjust the samples for differences in library size. Ribosomal genes and genes not expressed in the smallest group size minus one sample greater than one count-per-million were excluded from further analysis. The TMM size factors and the matrix of counts were then imported into the R/Bioconductor package Limma. Performance of the samples was assessed with Spearman correlations, a MultiDimensional Scaling plot, and hierarchical clustering. Weighted likelihoods based on the observed mean-variance relationship of each gene and sample were then calculated with the voomWithQualityWeights. The performance of all genes was assessed with plots of the residual standard deviation of each gene to their average log-count with a robustly fitted trend line of the residuals. Differential expression analysis was then performed to analyze differences among conditions, and the results were filtered for only those genes with Benjamini-Hochberg false-discovery rate (FDR) adjusted p-values less than or equal to 0.05.

### PCA plots and heatmaps

**Fig. 1:**
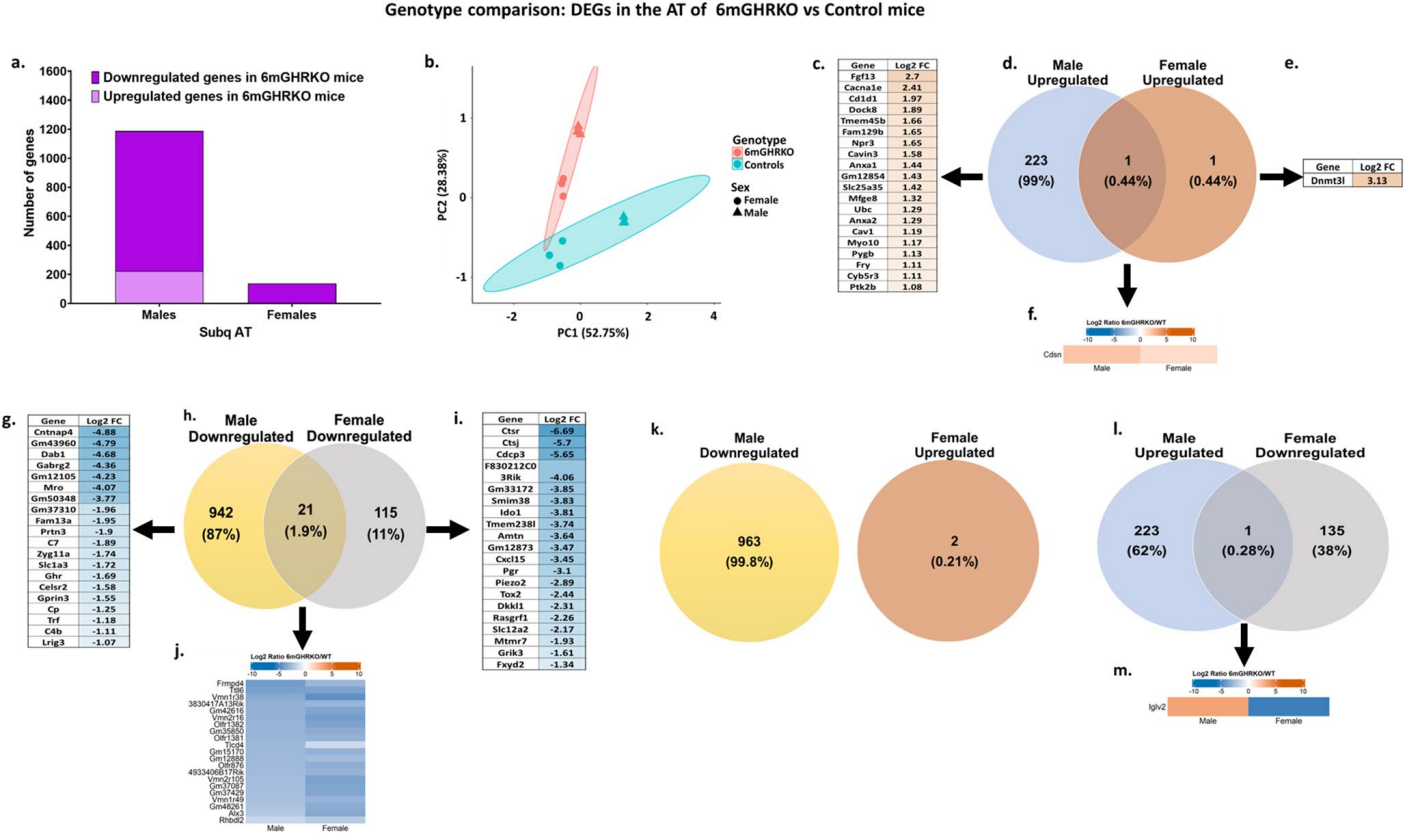
Total number of DEGs and unique, shared and divergent genes in the genotype comparison (6mGHKO vs. control mice).** (a)** Number of up- and down-regulated genes in the genotype contrast. **(b)** Principal component analysis (PCA) on the gene expression clustered by genotype. **(a)** Genes uniquely upregulated in the male 6mGHRKO vs. male control mice comparison. **(b)** Venn diagram of the upregulated genes of the male 6mGHRKO vs. male control mice and female 6mGHRKO vs. female control mice comparisons. **(c)** Genes uniquely upregulated in the male 6mGHRKO vs. male control mice comparison. **(d)** Venn diagram of the upregulated genes of the male 6mGHRKO vs. male control mice and female 6mGHRKO vs. female control mice comparisons. **(e)** Genes uniquely upregulated in the female 6mGHRKO vs. female control mice comparison. **(f)** Shared upregulated genes of the male 6mGHRKO vs. male control mice and female 6mGHRKO vs. female control mice comparisons. **(g)** Genes uniquely downregulated in the male 6mGHRKO vs. male control mice comparison. **(h)** Venn diagram of the downregulated genes of the male 6mGHRKO vs. male control mice and female 6mGHRKO vs. female control mice comparisons. **(i)** Genes uniquely downregulated in the female 6mGHRKO vs. female control mice comparison. **(j)** Shared downregulated genes of the male 6mGHRKO vs. male control mice and female 6mGHRKO vs. female control mice comparisons. **k.** Divergent genes of the downregulated genes in male 6mGHRKO vs. male control mice and the upregulated genes in female 6mGHRKO vs. female control mice comparisons. **l.** Venn diagram of the divergent genes of the upregulated genes in male 6mGHRKO vs. male control mice and the downregulated genes in female 6mGHRKO vs. female control mice comparisons. **m.** Divergent genes of the upregulated genes in male 6mGHRKO vs. male control mice and the downregulated genes in female 6mGHRKO vs. female control mice comparisons. 6mGHRKO, 6-month growth hormone receptor knockout

**Fig. 2:**
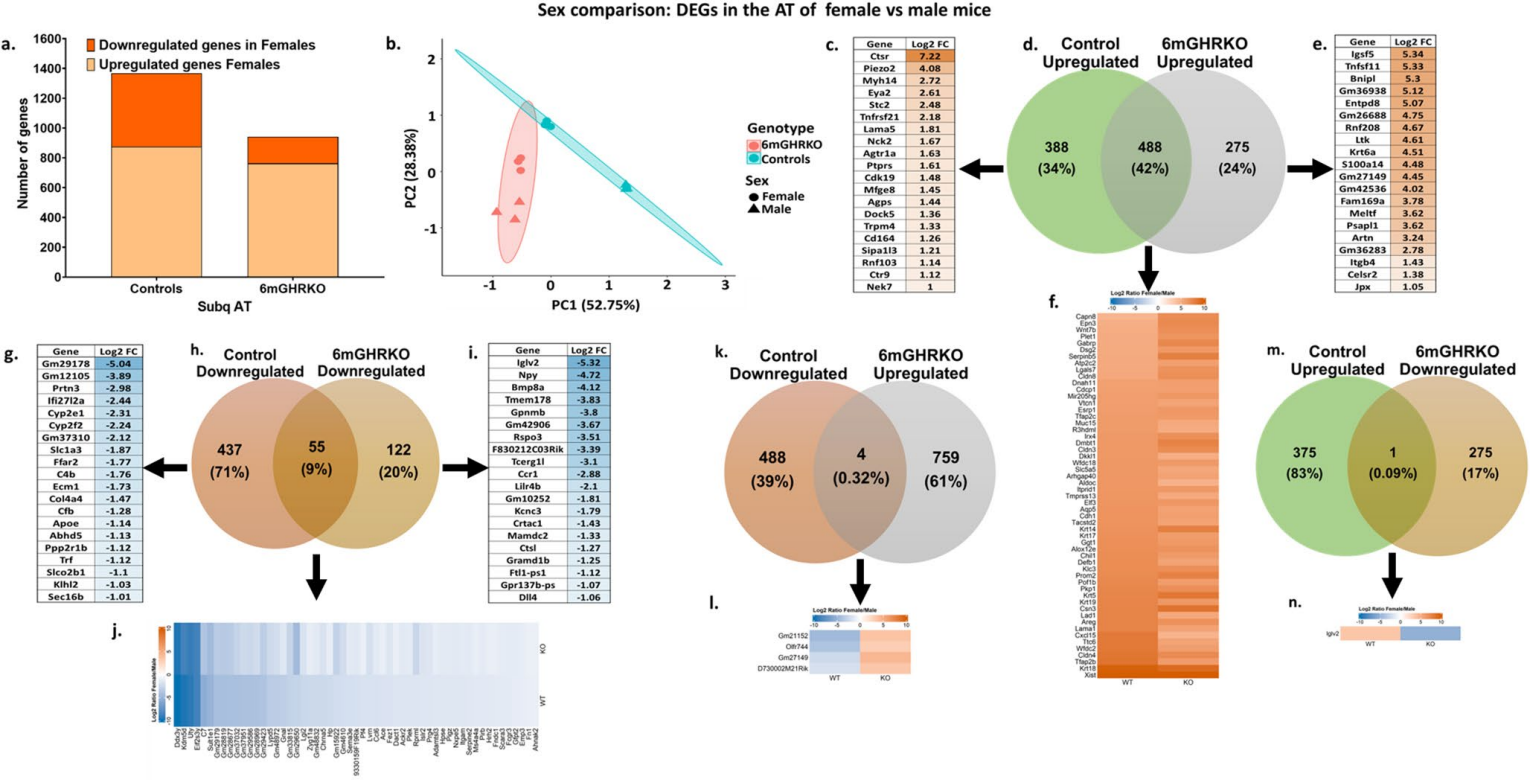
Total number of DEGs and unique, shared and divergent genes in the sex comparison (female vs. male mice). **(a)** Number of up- and down-regulated genes in the sex (female vs. male mice) contrast. **(b)** Principal component analysis (PCA) on the gene expression clustered by sex. **(c)** Genes uniquely upregulated in the in the female control vs. male control mice comparison. **(d)** Venn diagram of the upregulated genes of the female control vs. male control mice and male 6mGHRKO vs. female 6mGHRKO mice comparisons. **(e)** Genes uniquely upregulated in the in the female 6mGHRKO vs. male 6mGHRKO mice comparison. **(f)** Shared upregulated genes of the female control vs. male control mice and female 6mGHRKO vs. male 6mGHRKO mice comparisons. **(g)** Genes uniquely downregulated in the in the female control vs. male control mice comparison. **(h)** Venn diagram of the downregulated genes of the female control vs. male control mice and male 6mGHRKO vs. female 6mGHRKO mice comparisons. **(i)** Genes uniquely downregulated in the in the female 6mGHRKO vs. male 6mGHRKO mice comparison. **(j)** Shared upregulated genes of the female control vs. male control mice and female 6mGHRKO vs. male 6mGHRKO mice comparisons. **k.** Venn diagram of the divergent genes of the downregulated genes in female control vs. male control mice and the upregulated genes in female 6mGHRKO vs. female 6mGHRKO mice comparisons. **l.** Divergent genes of the downregulated genes in female control vs. male control mice and the upregulated genes in female 6mGHRKO vs. female 6mGHRKO mice comparisons. **m.** Venn diagram of the divergent genes of the upregulated genes in female control vs. male control mice and the downregulated genes in female 6mGHRKO vs. female 6mGHRKO mice comparisons. **n.** Divergent genes of the upregulated genes in female control vs. male control mice and the downregulated genes in female 6mGHRKO vs. female 6mGHRKO mice comparisons. 6mGHRKO, 6-month growth hormone receptor knockout

To visualize spatial differentiation among genotypes and sexes, R with RStudio was used with edgeR and limma libraries to create a principal component analysis (PCA). Non-zero values were filtered from the dataset, and then two standardized and centered PCAs were performed, clustered by genotype and sex separately, and graphed using ggplot2. The gene expression profiles in Figs. 1 and 2 were adapted from code published by Duncan et al. [19]. Heatmaps were generated using ComplexHeatmap [20].

### Gene ontology (GO), pathway analysis of DEGs and upstream transcriptional regulators

The gene ontology (GO; http://geneontology.org/) enrichment of DEGs was implemented using the hypergeometric test, in which p-value is calculated and adjusted as a q-value. Hierarchical GO terms with q < 0.05 were considered significantly enriched. Ingenuity Pathway Analysis (IPA) from QIAGEN was used to perform the functional enrichment pathway and upstream transcriptional regulator analysis https://www.qiagenbioinformatics.com/products/ingenuity-pathway-analysis/). All differentially expressed genes (log_2_ fold change > 1.5, FDR < 0.05) were used for the analysis. The z-score was used to infer the activation state which was calculated using the log2 fold change values of each gene.

## Results

### Reduced GH action at an adult age alters the AT gene expression of male mice more than female mice

Differentially expressed genes (DEGs) in AT from male and female mice were analyzed via RNA sequencing to compare 6-month-old GHR knockout (6mGHRKO) and control groups. Two main sets of comparisons were conducted: (i) genotype-based (male 6mGHRKO vs. male control; female 6mGHRKO vs. female control (Fig. 1a and l), and (ii) sex-based (control female vs. control male; 6mGHRKO female vs. 6mGHRKO male (Fig. 2a and l). The goal of the genotype comparison was to assess how adult-onset GHR deletion affects adipose gene expression, while the sex-based comparison focused on identifying sex-specific expression differences within the same genotype (Table 1).

**Table 1:** Comparisons used for the differentially expressed genes (DEG) analysis in subcutaneous adipose tissue

Analysis	Comparisons	#DEGs
Genotype	Male 6mGHRKO vs. Male control	1187
	Female 6mGHRKO vs. Female control	138
Sex	Male control vs. Female control	1368
	Female 6mGHRKO vs. male 6mGHRKO	940

The genotype contrast revealed more DEGs in the males than in the females. Specifically, 1187 DEGs (224 upregulated, 963 downregulated) were found in male 6mGHRKO mice versus controls, whereas female 6mGHRKO mice showed only 138 DEGs (2 upregulated, 136 downregulated), an 8.6-fold higher DEG count in males. In the sex comparisons, 1368 genes (876 upregulated, 492 downregulated) were differentially expressed between control females and males, compared to 940 genes (763 upregulated, 177 downregulated) in the 6mGHRKO female vs. male comparison, representing a 1.4-fold reduction in sex-associated DEGs following GHR deletion. Principal component analysis (PCA) illustrated clear segregation of groups based on both genotype and sex (Figs. 1b and 2b), highlighting distinct expression profiles.

### Predominance of downregulated genes following GHR deletion in adult mice

To further explore genotype-related gene expression differences in both sexes, DEGs were categorized into upregulated (Figs. 1c–f), downregulated (Figs. 1g–j), and divergent (Figs. 1k–m) subsets in 6mGHRKO relative to controls. The top 20 genes with the highest or lowest log_2_ fold change (LFC) values in 6mGHRKO males and females versus controls are displayed in Fig. 1c, e and g, and 1i, with complete gene lists, corresponding LFCs, and false discovery rates (FDR) detailed in Supplementary Tables 2–5.

In the genotype comparisons, male mice displayed a greater number and proportion of uniquely expressed genes in AT following GHR deletion than females. Specifically, 223 genes (representing 99% of all upregulated DEGs) were uniquely upregulated in males, whereas only one gene -Corneodesmosin, *Cdsn*- (0.44%) was uniquely upregulated in females (Fig. 1d; Suppl. Tables 2 and 3). Similarly, 942 genes (87% of downregulated DEGs) were exclusively downregulated in males, while females showed 115 unique downregulated genes (11%) (Fig. 1h; Suppl. Tables 4 and 5). Notably, only one upregulated gene (0.44% of the total) (Fig. 1b and f) and 21 downregulated genes (1.9%) (Fig. 1h and j) were common to both sexes. Interestingly, a single gene, Immunoglobulin Lambda Variable 2 (*Iglv2)*, displayed opposite regulation—upregulated in males and downregulated in females—following GHR ablation (Fig. 1l and m).

### Adult onset GHR ablation reduces the number of DEGs between males and females

To evaluate how GHR ablation affects sex-specific gene expression, DEGs in females versus males were analyzed within each genotype group (control and 6mGHRKO), focusing on upregulated, downregulated, shared, and divergent genes. Notably, the proportion of unique DEGs between female and male AT was lower in the 6mGHRKO group than in controls. Specifically, female controls showed 388 uniquely upregulated genes (34% of total upregulated DEGs), while 6mGHRKO females had 275 (24%) when compared to their respective male counterparts (Fig. 2a and d; Suppl. Tables 6 and 7). Similarly, female controls exhibited 437 unique downregulated genes (71% of total), compared to only 122 (20%) in 6mGHRKO females (Fig. 2a and h; Suppl. Tables 8 and 9). The top 20 DEGs exclusive to either control or 6mGHRKO females in comparison to males are highlighted in Fig. 2c, e and g, and 2i.

Among the DEGs shared between control and 6mGHRKO females versus males, 488 genes (42% of upregulated) and 55 genes (9% of downregulated) were commonly regulated (Fig. 2d, f and h, and 2j). To identify divergent expression patterns, DEG lists from both genotypes were compared (Fig. 2k and n). Only four genes showed inverse regulation—being downregulated in control females but upregulated in 6mGHRKO females when compared to males (Fig. 2k and l). Additionally, one gene, *Iglv2*, was uniquely upregulated in control females and downregulated in 6mGHRKO females (Fig. 2m and n).

### Gene ontology (GO) analysis of male and female 6mGHRKO mice and controls

**Fig. 3:**
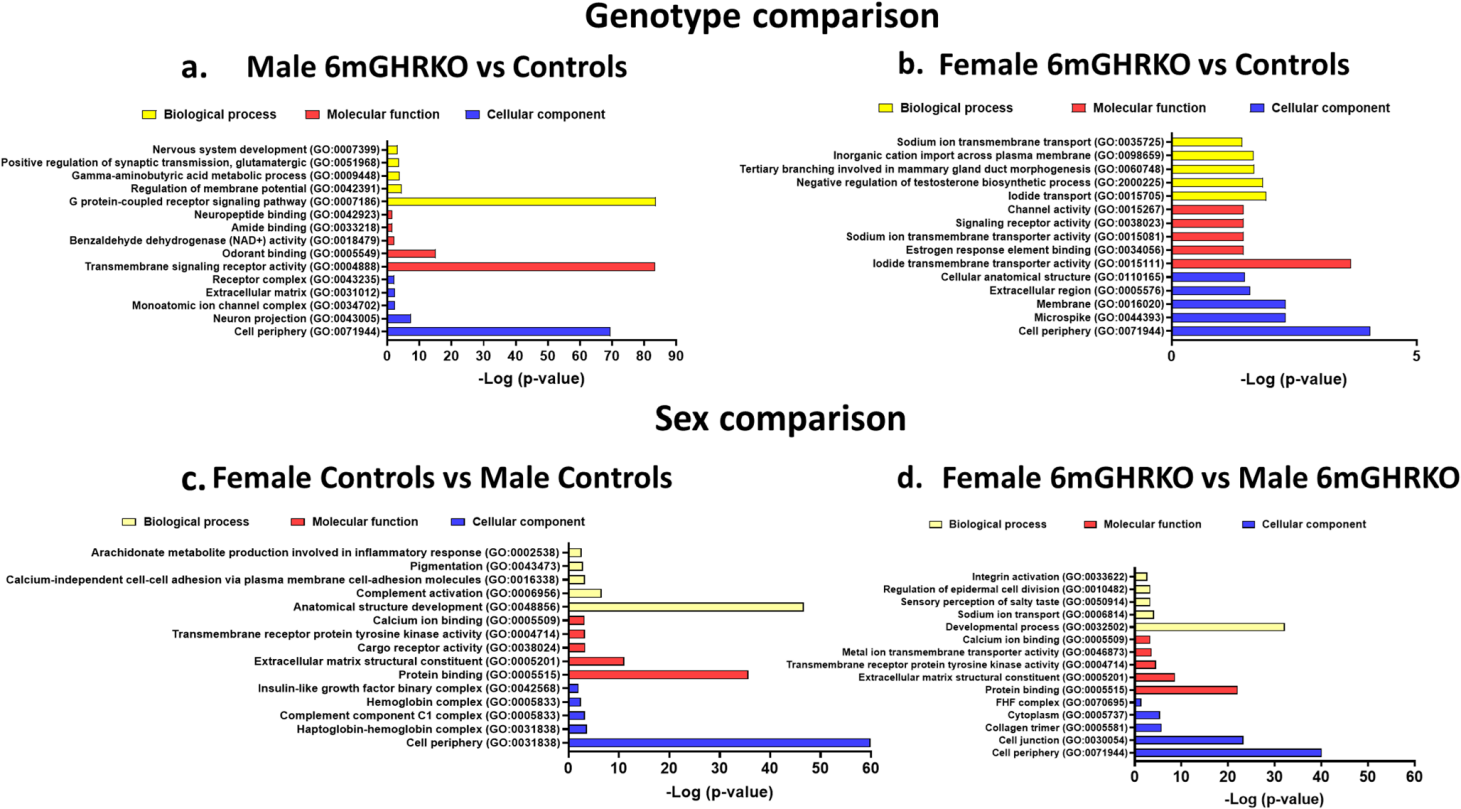
Gene ontology (GO) of for the genotype and sex comparisons.** (a)** GO of the DEGs of the male 6mGHRKO vs. male control mice comparison. **(b)** GO of the DEGs of the female 6mGHRKO vs. female control mice comparison. **(c)** GO of the DEGs of the female control vs. male control mice comparison. **(d)** GO of the DEGs of the female 6mGHRKO vs. male 6mGHRKO mice comparison. Bar graphs show the five most significantly enriched GO terms found in the biological process (yellow), molecular function (red) and cellular component (blue) categories. *P* < 0.05. 6mGHRKO, 6-month growth hormone receptor knockout

To assess the biological and functional implications of the differentially expressed genes (DEGs), we performed Gene Ontology (GO) enrichment analysis on the DEG datasets derived from both genotype and sex comparisons (Suppl. Tables 2–7). Genes were categorized under three GO domains: biological process, molecular function, and cellular component. Figure 3 highlights the top five enriched terms for each category across both comparisons.

In the comparison between male 6mGHRKO and control mice, the most significantly enriched terms were ‘G protein-coupled receptor signaling pathway’ under biological processes, and ‘Transmembrane signaling receptor activity’ under molecular functions (Fig. 3a, yellow and red bars). Conversely, the comparison of 6mGHRKO vs. control female mice yielded fewer DEGs (see Table 1), which translated to lower GO enrichment significance (x-axis, Fig. 3b). Notably, the enriched terms in this group were predominantly associated with ion signaling, including processes such as ‘Sodium ion transmembrane transport’, ‘Inorganic cation import across plasma membrane’, and ‘Iodide transport’, as well as related functions like ‘Sodium ion transmembrane transporter activity’ and ‘Iodide transmembrane transporter activity’ (Fig. 3b, yellow and red bars). Additionally, GO terms such as ‘Negative regulation of testosterone’ and ‘Estrogen response element binding’ were notably enriched in female samples.

In sex-based comparisons within genotypes, GO analysis revealed enrichment in processes related to extracellular matrix (ECM) remodeling, immune function, and ion transport in both control and 6mGHRKO mice. For control animals, prominent biological processes included ‘Anatomical structure development’ and ‘Complement activation’ (Fig. 3c, yellow bars), while enriched molecular and cellular components featured ‘Calcium ion binding’, Extracellular matrix structural constituent’, and ‘Complement C1 complex’ (Fig. 3c, red and blue bars). In the 6mGHRKO sex comparison, ‘Developmental process’ and ‘Protein binding’ emerged as the top enriched biological process and molecular function, respectively (Fig. 3d, red and blue bars). This group also displayed enrichment in ECM and ion-related terms such as ‘Sodium ion transport’, Calcium ion binding’, ‘Metal ion transmembrane transporter activity’, ‘Collagen trimer’, and ‘Cell junction’ (Fig. 3d). Notably, the term ‘Cell periphery’ was consistently the most enriched cellular component across all GO analyses (Fig. 3a–d, blue bars).

### Pathway analysis reveals a reduced activation of cellular pathways in 6mGHRKO male mice compared to controls

**Fig. 4:**
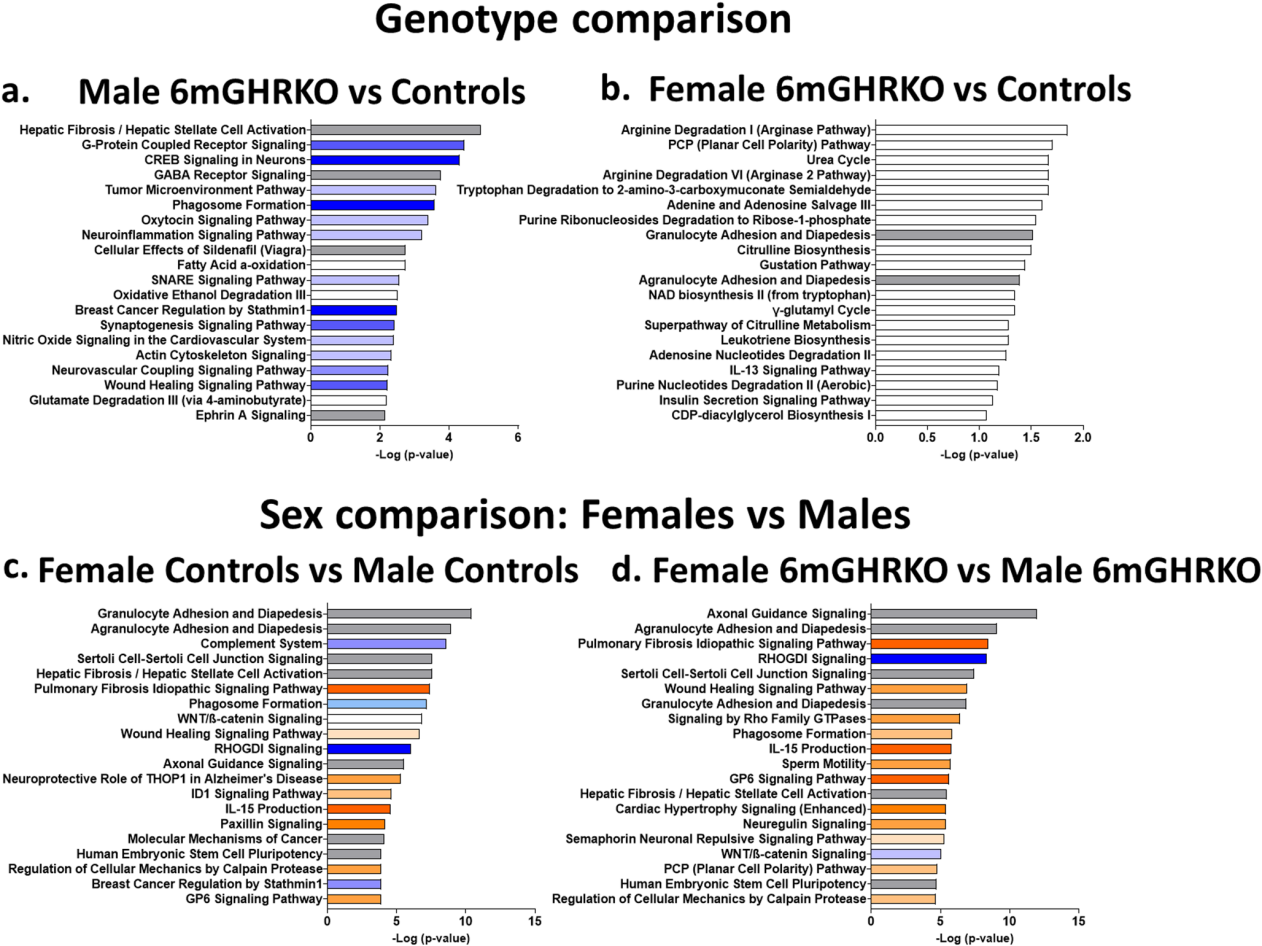
Ingenuity Pathway analysis (IPA) of the differentially expressed genes from the genotype and sex comparisons.** (a)** Pathway analysis of the DEGs of the male 6mGHRKO vs. male control mice comparison. **(b)** Pathway analysis of the DEGs of the female 6mGHRKO vs. female control mice comparison. **(c)** Pathway analysis of the DEGs of the female control vs. male control mice comparison. **(d)** Pathway analysis of the DEGs of the female 6mGHRKO vs. male 6mGHRKO mice comparison. Bar graphs show the 20 most altered biological pathways according to the P-value. Pathways were obtained IPA. Z scores were used to predict pathways that were activated (orange) or repressed (blue), grey bars indicate neutral z-score and white bars indicate pathways that were not classified

Pathway analysis was used to investigate the pathways that were significantly activated, repressed, or modulated by the DEGs of both genotype (Fig. 4a and b) and sex (Fig. 4c and d) pairwise comparisons. Interestingly, the pathways of the genotype comparison showed either a repressed or neutral status. Specifically, 14 out of the 20 most significant altered pathways showed a repressed status in the male 6mGHRKO mice when compared to controls (Fig. 4a). Furthermore, the suppressed pathways are associated with either extracellular matrix remodeling (ECM) and fibrosis or vesicle formation. For example, while the most significantly altered pathway was ‘Hepatic Fibrosis/Hepatic Stellate Cell Activation’, some of the pathways that showed repressed status and are associated with ECM remodeling are ‘Tumor Microenvironment Pathway’ and ‘Wound Healing Signaling Pathway’ (Fig. 4a). Similarly, the pathways that showed repressed status and are associated with vesicle formation are ‘Phagosome Formation’, ‘SNARE Signaling Pathway’, and ‘Actin Cytoskeleton Signaling’ (Fig. 4a). As expected, because of the small number of DEGs between female 6mGHRKO and control mice, the pathway analysis did not show a strong significance (-log (p-value)) or clear activation status. Nevertheless, 10 of the 20 most significantly altered pathways in 6mGHRKO female mice were associated with amino acid or nucleotide metabolism; some of these pathways are: ‘Arginine Degradation I (Arginase Pathway)’, ‘Urea Cycle Arginine Degradation VI (Arginase 2 Pathway)’, ‘Tryptophan Degradation to 2-amino-3-carboxymuconate Semialdehyde’, ‘Adenine and Adenosine Salvage III’, ‘Purine Ribonucleosides Degradation to Ribose-1-phosphate’, ‘NAD biosynthesis II (from tryptophan)’, ‘γ-glutamyl Cycle’, ‘Adenosine Nucleotides Degradation II’ and ‘Purine Nucleotides Degradation II (Aerobic)’ pathway (Fig. 4b).

### Females show a predominant activation of cellular pathways irrespective of genotype

Pathway analyses for the sex comparisons showed that female controls and female 6mGHRKO mice when compared to male controls and male 6mGHRKO, respectively, showed activation of pathways associated with ECM remodeling and fibrosis (Fig. 4c and d). That is, pathways such as ‘Pulmonary Fibrosis Idiopathic Signaling Pathway’, ‘Wound Healing Signaling Pathway’, ‘Hepatic Fibrosis/Hepatic Stellate Cell Activation’, ‘Cardiac Hypertrophy Signaling (Enhanced)’ and ‘Molecular mechanisms of cancer’ are made up mostly by genes involved in ECM remodeling (Fig. 4c and d). Other pathways that are also active in females compared to males are ‘IL15 production’ and ‘GP6 signaling pathway’, while ‘RhoGDI signaling’ pathway’ was repressed and’ WNT/B-catenin signaling’ and ‘Phagosome formation’ pathways were inversely active in female vs. male comparison in both genotypes (Fig. 4c and d).

### Alternate gene expression profile analysis showed enriched cluster in male mice

**Fig. 5:**
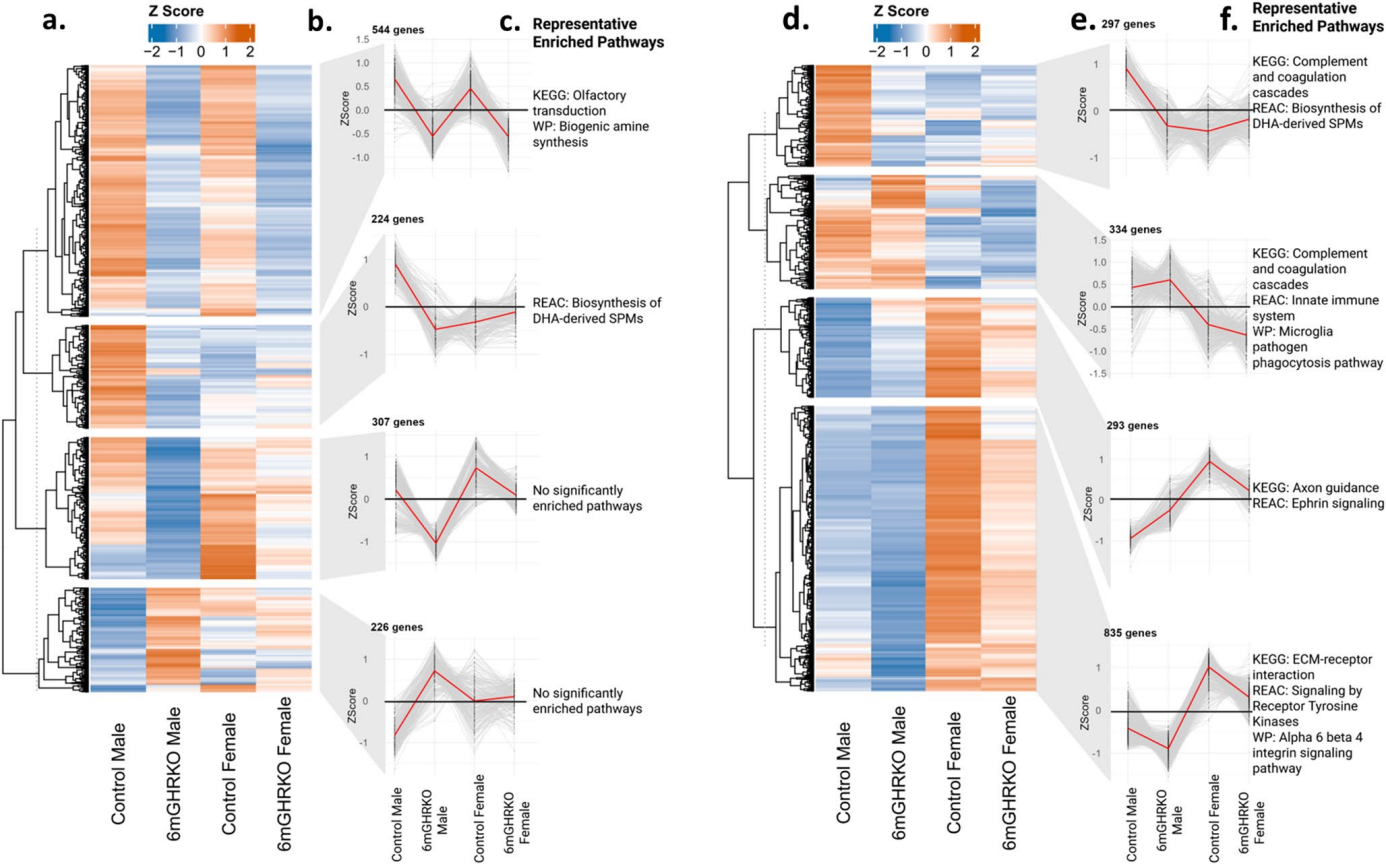
Alternate gene expression profile analyses of genotype- and sex-based comparisons in subcutaneous adipose tissue. a–c. Genotype comparison (control vs. 6mGHRKO within each sex). **(a)** Heatmap showing Z-score expression values of genes significantly altered in at least one genotype comparison, clustered into four expression pattern groups. **(b)** Average expression profiles of each gene cluster across treatment groups. **(c)** Representative pathways enriched within each cluster, highlighting functional differences between control and 6mGHRKO mice. **d–f.** Sex comparison (female vs. male within each genotype). **(d)** Heatmap showing Z-score expression values of genes significantly altered in at least one sex comparison, clustered into four expression pattern groups. **(e)** Average expression profiles of each gene cluster across groups. **(f)** Representative pathways enriched within each cluster, highlighting sex-dependent functional differences within control and 6mGHRKO mice. Together, these analyses illustrate that adult-onset GHR disruption results in distinct transcriptional clustering patterns across genotype and sex, with pathway enrichment analyses suggesting potential biological functions associated with each cluster

To further assess differential gene expression among the WT male, 6mGHRKO male, WT female, and 6mGHRKO female groups, we generated a gene expression profile highlighting genes significantly altered in either the control male vs. 6mGHRKO male comparison or the control female vs. 6mGHRKO female comparison (Fig. 5a). These genes were categorized into four distinct expression pattern groups (Fig. 5b), followed by overrepresentation analysis (ORA) to identify enriched biological functions. Representative pathways associated with each gene group are shown in Fig. 5c. We also performed differential gene expression for the sex comparison (females vs. males of controls and 6mGHRKOmice). We generated a gene expression profile highlighting genes significantly altered in either the control female s vs. control male comparison or the 6mGHRKO females vs. 6mGHRKO males comparison. These genes were categorized into four distinct expression pattern groups, followed by overrepresentation analysis to identify enriched biological functions. Representative pathways associated with each gene group are shown in Fig. 5.

In the alternate gene expression analysis on the genotype comparison, the largest cluster of genes (544 genes) had high expression in both control groups and low expression in both 6mGHRKO groups, and these genes were associated with the “olfactory transduction” and “biogenic amine synthesis” pathways. In contrast, a cluster of 244 genes that has high expression specifically in control males was associated with the “Biosynthesis of DHA-derived SPMs” pathway, while the other two clusters did not have significantly enriched pathways.

The alternate gene expression analysis on the significant genes from the sex comparison yielded a similar result, with a cluster of genes that are specifically high in control males being associated with the “Biosynthesis of DHA-derived SPMs” pathway, but also with the Complement and coagulation cascades” pathway. A cluster of 334 genes that had high expression in both male groups was also associated with the “Complement and coagulation cascades” pathway, but also with the “Innate immune system” and “Microglia pathogen phagocytosis” pathways. The two remaining clusters were high in both female groups, and were associated with “Axon guidance” and “Ephrin signaling” pathways or the “ECM receptor interaction”, “Signaling by receptor tyrosine kinases”, and “Alpha 6 beta 4 integrin signaling” pathways.

### Beta-estradiol and ECM-associated upstream regulators of gene expression are altered in females vs. males and with GHR ablation at an adult age

Ingenuity Pathway Analysis (IPA) was utilized to pinpoint upstream transcriptional regulators potentially responsible for the differential gene expression observed in both genotype and sex-based analyses. These upstream regulators are defined as molecules (such as transcription factors) that influence the expression of other genes. Table 2 highlights the top five most significantly impacted upstream regulators for each comparison. In the genotype analysis, progesterone emerged as the most significantly affected regulator in male 6mGHRKO mice compared to controls. In contrast, beta-estradiol exhibited a predicted inhibition pattern in female 6mGHRKO mice relative to controls. Notably, beta-estradiol also stood out in the sex-based comparison, showing a consistent activation in females regardless of genotype. Additionally, several of the regulators predicted to differ between sexes—such as TGFB1, TNF, and ADAMTS18—are linked to extracellular matrix (ECM) remodeling processes (Table 2).

**Table 2:** Top five predicted upstream regulators using ingenuity pathway analysis (IPA). The upstream regulators analysis is based on prior knowledge of expected effects between transcriptional regulators and their target genes

	Upstream Regulator	Molecule Type	Predicted Activation	Activation z-score	*p*-value of overlap
Male 6mGHRKO vs. male controls	Progesterone	chemical – endogenous mammalian		1.361	5.17E-07
	SNCA	Enzyme		−0.951	6.80E-07
	Thioacetamide	chemical toxicant		1.583	7.47E-07
	CREB1	Transcription regulator		1.275	1.12E-06
	SOD1	enzyme			3.36E-06
Female 6mGHRKO vs. female controls	CAV1	transmembrance receptor			1.12E-07
	beta-estradiol	chemical – endogenous mammalian	Inhibited	−3.326	3.42E-06
	CDK8	kinase			2.22E-05
	EIF4G2	translation regulator			6.93E-05
	diethylstilbestrol	chemical drug		−0.285	1.46E-04
Female controls vs. male control	beta-estradiol	chemical – endogenous mammalian	Activated	2.819	1.52E-37
	dexamethasone	chemical drug		0.679	1.84E-29
	TGFB1	growth factor		0.083	6.03E-26
	progesterone	chemical – endogenous mammalian		1.901	3.03E-25
	TNF	cytokine		0.445	3.73E-24
Female 6mGHRKO vs. male 6mGHRKO	beta-estradiol	chemical-endogenous mammalian	Activated	4.57	5.06E-28
	ADAMTS18	peptidase	Activated	4.257	6.98E-27
	estrogen receptor	group	Activated	2.902	8.03E-19
	TP63	transcription regulator		0.915	4.8E-17
	TGFB1	growth factor		1.193	5.79E-17

## Discussion

GH plays a key role in various physiological and cellular processes, including skeletal growth, regulation of lipid, glucose, and protein metabolism, extracellular matrix (ECM) remodeling, aging, and cancer development [2, 21]. In addition to promoting IGF-1 synthesis, GH exerts IGF-1 independent effects such as anti-insulin effects, in part due to its capacity to stimulate lipolysis in AT, leading to elevated circulating free fatty acids that interfere with insulin signaling and secretion [20, 22]. Beyond promoting fat breakdown, GH influences the overall structure and function of AT [23], affecting parameters such as tissue mass [24], lipid metabolism, adipocyte size, stromal vascular composition, immune cell infiltration, accumulation of senescent cells [25], and ECM organization. Our previous work demonstrated that mice with GH resistance induced at a mature adult stage (6mGHRKO mice) exhibit lower IGF-1 levels along with decreased lipid and protein oxidative damage [26]. These mice also show improved aging phenotypes, with extended lifespan in females and enhanced insulin sensitivity and cancer resistance in males [26]. Given that subq AT is highly sensitive to GH, we hypothesized that sex-specific transcriptional changes in AT might contribute to the observed benefits in 6mGHRKO mice. To explore this, we employed RNA sequencing (RNAseq) with two primary aims: (i) to assess how GH receptor deletion in adult mice alters adipose gene expression in each sex (genotype comparison), and (ii) to identify sex-related gene expression differences within each genotype (sex comparison).

When analyzing the number of differentially expressed genes (DEGs; log_2_ fold change >1.5, FDR < 0.05) in the genotype comparison (6mGHRKO vs. controls), we observed that male mice exhibited approximately 8.6 times more total DEGs than females. Additionally, males showed a greater number of uniquely expressed genes relative to their control counterparts, as opposed to DEGs shared between male and female 6mGHRKO mice. This suggests that the reduction of GH action influences gene expression in a sex-specific manner, with a more pronounced effect in males. An important observation in this study is the striking disparity in the number of differentially expressed genes (DEGs) between males and females following adult-onset GHR disruption. One possible explanation is biological: previous studies have shown that GH exerts stronger effects in male adipose tissue mass compares to females [27], which may account for the more robust transcriptomic response in males. Another consideration is technical: with a relatively small sample size (*n* = 3 per group), smaller effect sizes or greater variability in females may limit statistical power to detect changes at the transcript level. Thus, the lower number of DEGs observed in females should not be interpreted as absence of effect, but rather as reduced resolution under the current experimental design. Future studies with larger cohorts and complementary validation approaches will be needed to fully characterize the female-specific transcriptional responses to reduced GH action.

Notably, the majority of DEGs identified in both sexes were downregulated, indicating that GH ablation during adulthood predominantly suppresses gene expression. This result contrasts with the DEGs seen in mice with excess GH action (bGH transgenic mice) which showed a similar quantity of up- and down- regulated genes in the subq AT depot of male mice compared to controls [28]. Of note, most of the DEGs in the genotype comparison were downregulated, this aligns with the fact that the only upregulated gene in the females 6mGHRKO vs. controls comparison was Dnmt3l - a DNA methyltransferase-like protein that enhances methyltransferase activity and may contribute to gene repression through epigenetic regulation [29]. Interestingly, only male 6mGHRKO mice showed a decrease in *Ghr* gene expression in the RNAseq analysis. This was unexpected, as previous real-time qPCR experiments revealed significant reductions in *Ghr* expression in the subq AT of both sexes [26]. The discrepancy may stem from methodological differences— the RNAseq analysis used for this experiment involves mapping short reads to single reference genomes. Because the 6mGHRKO mice are generated by eliminating only part of the exon 4 of the *Ghr* gene, fragments of sequenced cDNA from elsewhere in the *Ghr* gene were aligned to the mouse reference genome leading to a similar number of counts of *Ghr* gene expression in knockouts and controls (Suppl. Figure. 1). On the other hand, qPCR targets a specific part of the *Ghr* gene using primers, one of which, in our case, binds to exon 4. Therefore, qPCR is able to detect this gene alteration better than standard RNAseq processing.

Because the longevity and metabolic benefits seen in 6mGHRKO mice are sex dependent, we also analyzed the DEG list of females vs. males comparison within genotype. We found that ablation of *Ghr* gene in the AT resulted in a 1.4-fold decrease in the number of total DEG when comparing females vs. males, indicating that the difference in gene expression between sexes is higher in the AT of normal mice than in the AT of 6mGHRKO mice. Furthermore, this suggests that reduced GH action at an adult age causes gene expression between males and females to become similar to each other. These gene expression phenomena have been shown in other studies, particularly in liver. Previous RNAseq analysis showed that the difference in gene expression between sexes in the liver was reduced in 6mGHRKO mice vs controls [30]. Furthermore, other reports also show that modulation of GH action reduces the sex-specific differences in gene expression. For example, GH ablation in rats due to hypophysectomy results in 90% reduction of the sex- specific differences in the liver [31]. In fact, male mice subject to continuous GH infusion show feminization’ of male liver gene expression wherein 86% of male-biased genes were repressed and 68% of female-biased genes were induced [32]. In the same line, upstream regulator analysis show that when comparing females vs. males reduction of GH action at an adult age resulted in predicted activation of upstream regulators that may drive sex-specific differences such as beta-estradiol and estrogen receptor.

Upstream regulator analysis also showed that the sex comparison had several upstream regulators associated with ECM remodeling processes and fibrosis (TGFB1, TNF, and ADAMTS18). These results align with the GO ontology and the IPA analysis, which highlighted activation of biological processes and pathways associated with ECM remodeling and immune cell infiltration when comparing females vs. males in control and 6mGHRKO mice. For example, pathways that are made up mostly by genes involved in ECM remodeling such as ‘Pulmonary Fibrosis Idiopathic Signaling Pathway’, ‘Wound Healing Signaling Pathway’, ‘Hepatic Fibrosis/Hepatic Stellate Cell Activation’, ‘Cardiac Hypertrophy Signaling (Enhanced)’ had a predicted activation in females compared to male mice. Furthermore, for the genotype comparison 14 out of the 20 most significant altered pathways had a predicted repressed status male 6mGHRKO mice when compared to controls. The pathways that showed suppression were also associated with ECM remodeling (‘Hepatic Fibrosis/Hepatic Stellate Cell Activation’, Tumor Microenvironment Pathway’ and ‘Wound Healing Signaling Pathway’). To note, genes that comprise these pathways had a clear distinction between the sex and genotype comparisons, where the sex comparison had upregulation of collagen and metalloproteinase genes in females vs. males (Col4a6, Col17a1, Col4a, Col16a1, Lama1, Mmp15, Tgfb3), while male 6mGHRKO mice had more downregulation of collagen subunits when compared to control mice (Col 19a1, Col26a1, Mmp9, Col4a3) (Suppl. Figure 2). Females 6mGHRKO did not show a clear trend due to the low number of DEGs seen in this comparison. there was a clear distinction in the sex and genotype comparison. These align with previous reports in mice and humans that show, in general, reduced GH action ameliorates collagen deposition, while increase GH action promotes ECM remodeling in AT and other tissues [33–36]. Altogether, these results suggest that ECM remodeling and fibrotic pathways -and genes- are more active in the AT of females vs. males. However, this activation seems to be reduced when GH action is decreased at an adult age.

In summary, we investigated differential gene expression, biological processes, signaling pathways, and potential upstream regulators in the Subq AT of male and female 6mGHRKO mice compared to controls (genotype comparison), as well as between sexes within each genotype (sex comparison). Taken together, our results show that adult-onset disruption of the GHR is associated with widespread transcriptional changes, particularly in males, and alters sex-specific differences in adipose tissue. These findings provide preliminary insight into molecular pathways, such as ECM remodeling, that may underline the healthspan benefits of reduced GH action. Importantly, these associations require further experimental follow-up before mechanistic conclusions can be drawn. That is, although our study provides novel insight into the transcriptional changes in subq AT following adult-onset GHR disruption, there are several limitations that must be acknowledged. First, the relatively small sample size (*n* = 3 per group) restricts the statistical resolution, particularly for detecting subtle changes in female mice. Second, our analyses are limited to transcriptomic data; we did not validate gene expression changes at the protein level or through histological approaches such as collagen staining. As such, the findings presented here should be interpreted as hypothesis-generating and require further experimental validation. In addition, our work focused exclusively on subq AT collected in 12 mo- adult-onset disrupted-GHR mice, and it remains possible that other adipose depots or timepoints may reveal distinct transcriptional responses. Despite these limitations, the data presented provide a valuable framework for understanding how reduced GH action at an adult age influences adipose tissue biology in a sex-dependent manner and highlight pathways that warrant future mechanistic investigation.

## Supplementary Information

Below is the link to the electronic supplementary material.

Supplementary Material 1 (DOCX 24.0 KB)

Supplementary Material 2 (DOCX 380 KB)

Supplementary Material 3 (XLSX 32.0 KB)

Supplementary Material 4 (XLSX 16.0 KB)

Supplementary Material 5 (XLSX 76.0 KB)

Supplementary Material 6 (XLSX 24.0 KB)

Supplementary Material 7 (XLSX 76.0 KB)

Supplementary Material 8 (XLSX 68.0 KB)

Supplementary Material 9 (XLSX 48.0 KB)

Supplementary Material 10 (XLSX 28.0 KB)

## Data Availability

The datasets generated and analyzed during the current study are available upon request. Our studies do not include the use of custom code or mathematical algorithms.

## Declarations

### Permission to reproduce material from other sources

NA.

### Clinical trial registration

NA.
