# Supplementary material for: Sex-Specific transcriptomic changes in adipose tissue following adult-onset disruption of growth hormone receptor
Source: Pituitary. 2025 Dec 5;29(1):5. doi: 10.1007/s11102-025-01603-3 (PMC12680793; doi:10.1007/s11102-025-01603-3)
Supplement: Supplementary file 1 — Supplementary Material 1 (DOCX 24.0 KB) [file 11102_2025_1603_MOESM1_ESM.docx]

**Suppl. Figure 1. Short Fragments align to exon 4 of the *Ghr* gene.** IGV Image showing the read coverage of the portion of the mouse *Ghr* gene that is disrupted in this experiment. Exon 4 has lower coverage in the 6mGHRKO animals, demonstrating that the gene has been disrupted. The coverage in other exons is normal, which causes *Ghr* expression to appear normal in the RNAseq analysis.


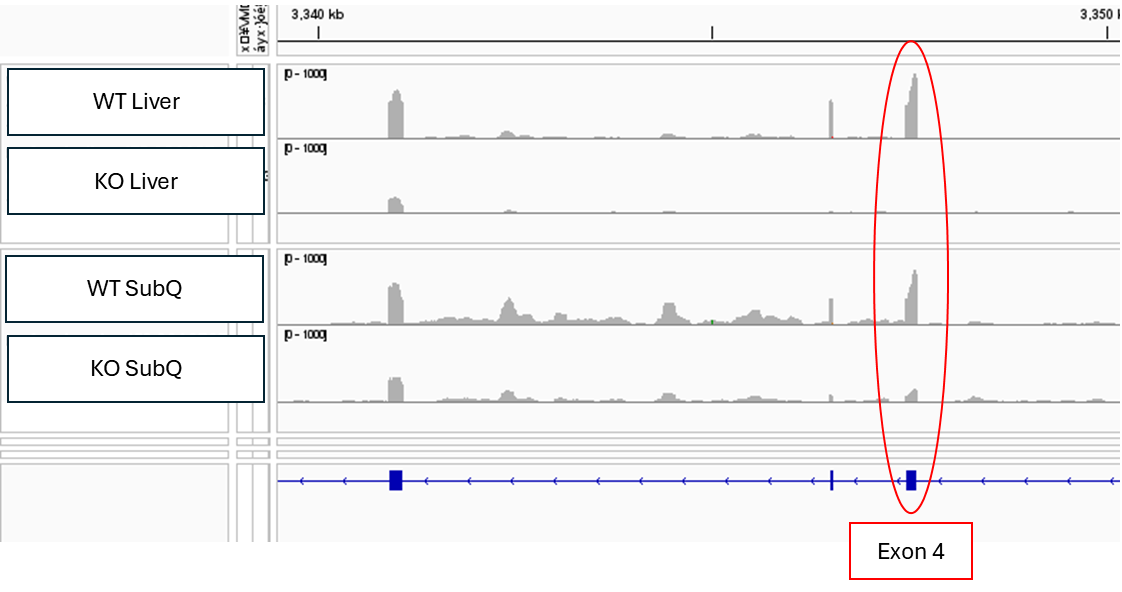

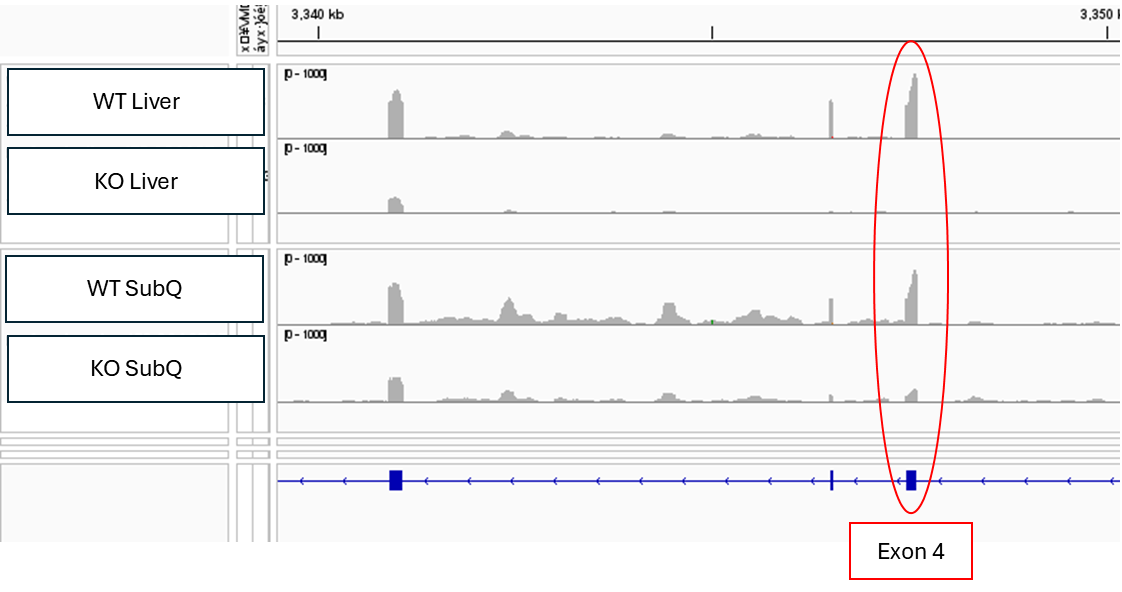


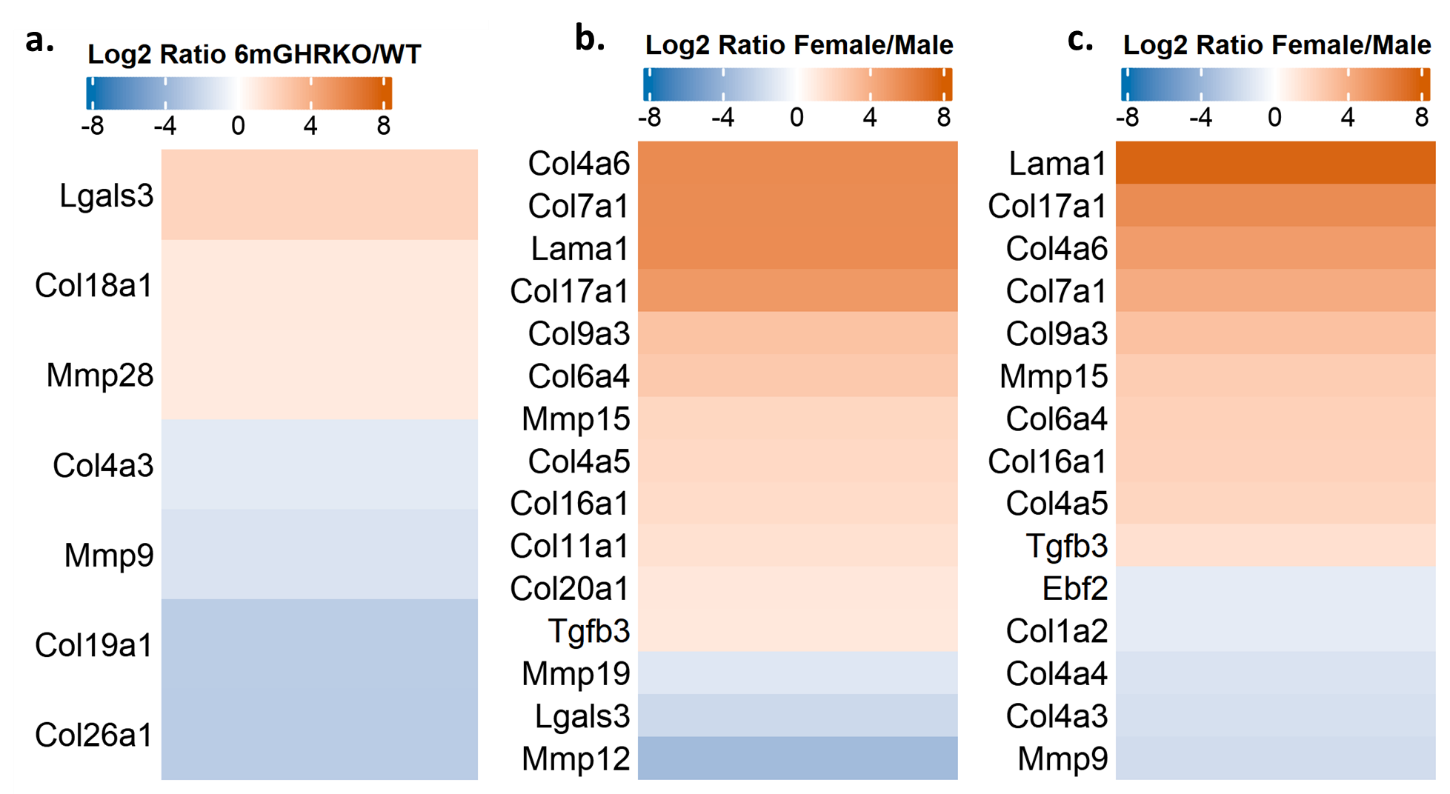


**Suppl. Figure 2. Gene expression of the Extracellular matrix (ECM) associated genes. a.** Gene expression of ECM genes in the male 6mGHRKO mice vs controls. **b.** Gene expression of ECM genes in the female control mice vs. male controls. **c.** Gene expression of ECM genes in the female 6mGHRKO mice vs. male 6mGHRKO mice.
