# Supplementary material for: Sex-Specific transcriptomic changes in adipose tissue following adult-onset disruption of growth hormone receptor
Source: Pituitary. 2025 Dec 5;29(1):5. doi: 10.1007/s11102-025-01603-3 (PMC12680793; doi:10.1007/s11102-025-01603-3)
Supplement: Supplementary file 2 — Supplementary Material 2 (DOCX 380 KB) [file 11102_2025_1603_MOESM2_ESM.docx]

**Suppl. Table 1. Body length and composition of 6mGHRKO mice at the time of dissection.** Nuclear Magnetic Resonance (NMR) measurements showed increased fat mass in 6mGHRKO compared to control mice at 12 months old. . Body length was not change.

| **Mouse #** | **Sex** | **Genotype** | **Length (cm)** | **Subq (g)** | **Fat (g)** | **Fat %** | **Weight (g)** |
| --- | --- | --- | --- | --- | --- | --- | --- |
| 156 | F | aoGHRKO | 10.3 | 1.475 | 7.8 | 25.7 | 30.46 |
| 168 | F | aoGHRKO | 9.6 | 0.983 | 5 | 19.9 | 25.19 |
| 170 | F | aoGHRKO | 9.4 | 1.507 | 8.5 | 28.9 | 29.52 |
| 139 | F | WT | 9.5 | 0.462 | 4 | 14.8 | 27.28 |
| 177 | F | WT | 10.2 | 0.377 | 4.8 | 16.1 | 30 |
| 125 | F | WT | 9.8 | 0.921 | 6.8 | 21.2 | 31.84 |
| 178 | M | aoGHRKO | 10.2 | 1.096 | 8.1 | 25.9 | 31.39 |
| 180 | M | aoGHRKO | 10 | 1.266 | 9.5 | 25.8 | 36.81 |
| 194 | M | aoGHRKO | 10 | 1.482 | 9.8 | 24.9 | 39.42 |
| 128 | M | WT | 9.9 | 0.336 | 2.6 | 7.9 | 32.83 |
| 120 | M | WT | 9.7 | 0.405 | 9 | 19.9 | 45.13 |
| 130 | M | WT | 9.8 | 0.432 | 3.1 | 9 | 34.74 |
